# The characteristics of Guillain–Barre syndrome in children in pre‐COVID‐19 and during the COVID‐19 pandemic: A cross‐sectional study

**DOI:** 10.1002/hsr2.1782

**Published:** 2023-12-21

**Authors:** Sepide Javankiani, Amir Nasrollahizadeh, Behdad Gharib, Morteza Heidari, Sara Memarian

**Affiliations:** ^1^ Children's Medical Center Tehran University of Medical Sciences Tehran Iran

**Keywords:** acute inflammatory demyelinating polyneuropathy, acute motor axonal neuropathy, COVID‐19, Guillain–Barre syndrome, neutrophil‐lymphocyte ratio

## Abstract

**Background and Aims:**

In the pathophysiology of Guillain–Barre syndrome (GBS), inflammation and immunity are believed to play a key role. The neutrophil‐lymphocyte ratio (NLR), monocyte‐lymphocyte ratio (MLR), and platelet‐lymphocyte ratio (PLR) have been recently identified as potential markers of inflammation or immunity. This study aimed to investigate whether NLR, MLR, and PLR are associated with GBS characteristics in children. We also assessed the impact of the COVID‐19 pandemic on the characteristics of GBS in Iran.

**Methods:**

In this retrospective cross‐sectional study, we reviewed the records of all 150 children diagnosed with GBS in the Children's Medical Center hospital affiliated with Tehran University of Medical Sciences (TUMS) from March 2017 until March 2022. The TUMS research ethics committee approved the study (Ethics code: IR.TUMS.CHMC.REC.1399.125). Patients' data including gender, age, clinical symptoms, laboratory findings, and electrodiagnostic study results were collected and analyzed.

**Results:**

This study involved 150 children, comprising 93 boys and 57 girls, with an average age of 7.53 ± 3.75 years. The analysis demonstrated that the number of hospitalization days increased with an increase in NLR (*p* = 0.025). Moreover, patients with abnormal electrodiagnostic study patterns had a higher risk of intensive care unit (ICU) admission (*p*: 0.027), although according to binary logistic regression, respiratory failure at admission time was the only significant factor increasing the risk of ICU admission (*p* = 0.035). The study also found that the pandemic has resulted in a shift from acute inflammatory demyelinating polyneuropathy to acute motor axonal neuropathy as the most common EMG‐NCV pattern in our patients (*p* < 0.001).

**Conclusion:**

We found that higher NLR was associated with a longer hospitalization duration and could potentially distinguish between severe and mild cases of GBS. We have also shown that the COVID‐19 pandemic has changed our patients' most frequent electromyography and nerve conduction velocity (EMG‐NCV) patterns.

## 1 INTRODUCTION

Guillain–Barre syndrome (GBS) is a group of diseases with peripheral neuropathy leading to acute neuromuscular failure. The etiology of this syndrome is probably due to an immune system attack on peripheral nerves.^1^ One of the most common causes of acute paralysis among children is GBS (49%), and its annual incidence in children is approximately 0.69 per 100,000.^2^ Guillain–Barre syndrome usually happens after a viral infection such as an upper respiratory tract infection or gastroenteritis.^3^

Guillain–Barre syndrome can be divided into at least four subgroups (from most to least prevalent): Acute inflammatory demyelinating polyneuropathy (AIDP), acute motor axonal neuropathy (AMAN), acute motor and sensory axonal neuropathy (AMSAN), and Miller‐Fischer syndrome (MF).^4^

Standard criteria for the diagnosis of Guillain–Barre syndrome were first published in 1978 by the National Institute of Neurological and Communicative Disorders (NINCDS). Clinical and Paraclinical features such as progressive motor weakness, areflexia, symmetry of symptoms, mild sensory symptoms, and increased protein level without increased WBC in cerebrospinal fluid (CSF) and slow or blocked nerve conduction pattern in electromyography and nerve conduction velocity (EMG‐NCV) were included in Asbury and colleagues criteria.^5^, ^6^

Guillain–Barre syndrome usually appears with numbness and weakness in the lower limbs. The symptoms often ascend to the upper parts of the body in a short time. Sometimes the weakness is so severe that the patient cannot walk, which may lead to complete paralysis. There is no accurate and definitive test to diagnose GBS, but the diagnosis is mostly based on symptoms such as muscle weakness, inability to walk, and preceding events. EMG‐MCV and CSF analysis are complementary tests to diagnose and determine the prognosis of GBS.^7^

The treatment mainly includes intravenous immunoglobulin (IVIG) injection and plasmapheresis.^8^

Factors such as white blood cells (WBC), erythrocyte sedimentation rate (ESR), C‐reactive protein (CRP), and acute phase reactant proteins such as albumin are widely used as inflammatory markers. Several studies have investigated the efficacy of new parameters, for instance, neutrophil‐lymphocyte ratio (NLR), platelet‐lymphocyte ratio (PLR), and monocyte‐lymphocyte ratio (MLR) in determining the prognosis of different conditions including neurological,^9^, ^10^, ^11^, ^12^, ^13^, ^14^ vascular diseases,^15^, ^16^ diabetes,^17^ and cancers.^18^ Furthermore, it has been shown that NLR can be used as a prognostic factor in GBS.^11^, ^12^, ^13^, ^19^

Due to the development of new inflammatory factors in establishing different aspects of inflammatory diseases, the current study is designed to figure out the efficacy of MLR, NLR, and PLR in determining the prognosis of children with different subtypes of GBS.

## 2 METHODS

This cross‐sectional study was conducted on patients with clinical and paraclinical diagnosis of GBS, hospitalized at Children's Medical Center Hospital, Tehran, Iran. All the GBS patients referred to the Children's Medical Center hospital (Neurology clinic and the Emergency department), affiliated to Tehran University of Medical Sciences (TUMS) from March 2017 to March 2022 have been included. The TUMS research ethics committee approved the study (Ethical code: IR.TUMS.CHMC.REC.1399.125).

The data were collected from their medical files. Variables including age, sex, length of hospitalization, need for intensive care unit (ICU) admission, clinical symptoms (paralysis of lower and upper limbs, inability to walk, weakness and fatigue, and respiratory failure) laboratory findings on the first day of admission (Complete blood count [CBC], ESR, CRP cerebrospinal fluid [CSF] analysis) were recorded and NLR and PLR, MLR were calculated.

Patients with a history of chronic diseases such as metabolic, malignant, other neurologic, or endocrinological diseases were excluded.

The first confirmed COVID‐19 case in Iran was reported on February 19, 2020.^20^ We divided our patients into two groups based on admission date: before and after COVID‐19 pandemic in Iran. Confirmation of COVID‐19 infection was assessed via a polymerase chain reaction test. The results of the electrodiagnostic study of patients were grouped into AIDP, AMAN, and AMSAN.

A specific finding in Guillain–Barre patients regarding cerebrospinal fluid analysis is the albuminocytological dissociation (ACD) pattern, which is characterized by elevated protein levels and normal WBC counts when examining CSF fluid. Our research has assumed this pattern as WBC ≤ 6 per cubic millimeter (mm^3^) and protein >40 milligrams per deciliter (mg/dL).

All data were analyzed by SPSS version 22. Descriptive and Frequency statistical tests were used to determine the prevalence of variables. The normality of the distribution of continuous variables was assessed via the Kolmogorov–Smirnov test. Independent *T* test and *χ* ^2^ were used subsequently to compare quantitative and normally distributed qualitative variables respectively, also Mann–Whitney *U* test and Kruskal–Wallis *H* test were applied for non‐normally distributed variables. Finally, logistic regression was performed to assess the prognosis. Statistical significance was set at *p* < 0.05.

## 3 RESULTS

**Figure 1:**
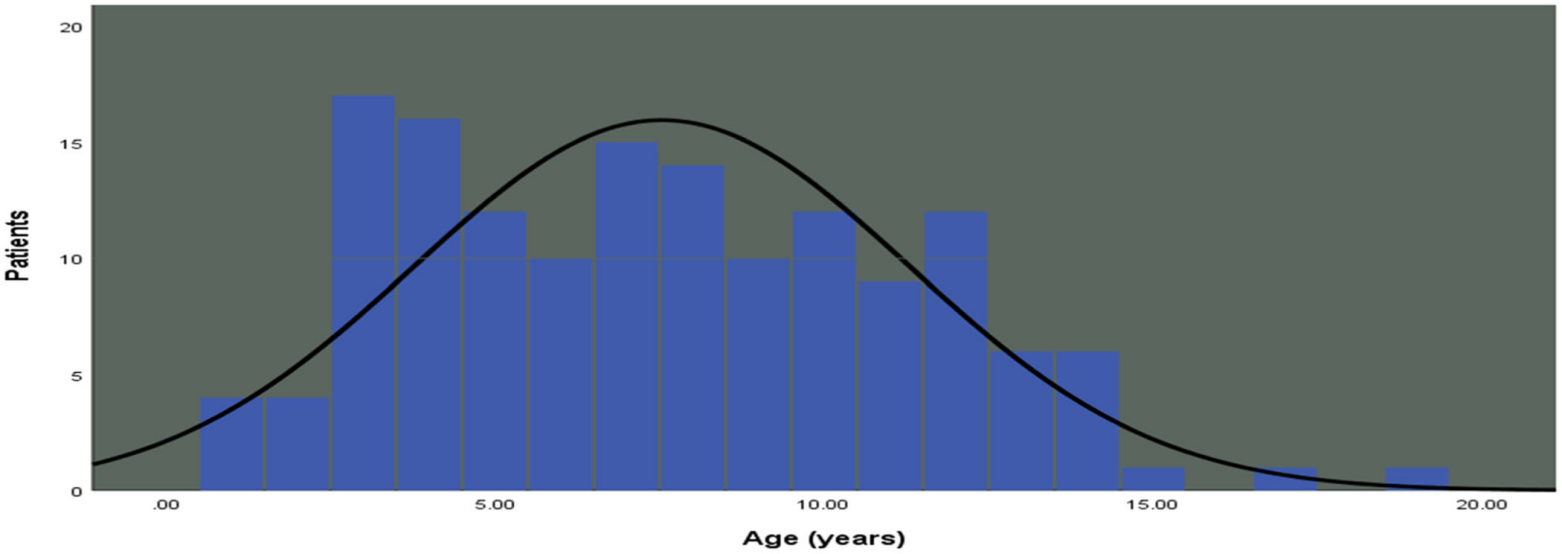
Age distribution of 150 children with Guillain–Barre syndrome.

One hundred and fifty children including 93 boys and 57 girls were included in this study. The mean age of the participants was 7.5 years (Figure 1). Clinical presentations in order from the most frequent to least were lower limb paralysis, inability to walk, upper limb paralysis, and respiratory failure. More than 50% of the patients reported preceding viral infection symptoms before the onset of GBS and 3% of patients have been diagnosed with COVID‐19. Also, among 150 Guillain–Barre patients, 117 received IVIG treatment and 8 children underwent plasmapheresis. No mortality was reported during hospitalization, but 38 people needed ICU admission. The average length of hospitalization was approximately 7.2 days. Demographic, clinical, and paraclinical characteristics of patients are shown in Table 1.

**Table 1:** Demographic and clinical and paraclinical results of patients.

		Total number	Mean (SD) or Number(%)	
Age (years)		150	7.53 ± 3.75	
Sex	Male	93	61.6%	
	Female	57	37.7%	
Plasmapheresis		150	8 (5.3%)	
IVIG		150	117 (67.8%)	
ICU admission		150	38 (25.2%)	
Hospital stays		150	7.23 ± 7.54 days	
Respiratory failure	Male	93	6 (4%)	*p* Value = 0.141
	Female	57	0 (0%)	
Upper limb paresis	Male	93	49(32.7%)	*p* Value = 0.335
	Female	57	26 (17.3%)	
Lower limb paresis	Male	93	88(58.7%)	*p* Value = 0.261
	Female	57	56(37.3%)	
Inability to walk	Male	93	87 (58%)	*p* Value = 0.734
	Female	57	54 (36%)	
COVID‐19 infection		150	5 (3.3%)	
Antecedent viral infection		150	89 (58.9%)	
CSF protein (mg/dL)		46	85.85 ± 66.23	
CSF glucose (mg/dL)		46	56.39 ± 12.68	
CSF WBC (per mm^3^)		46	5.26 ± 12.12	
WBC (per mm^3^)		148	9.44 ± 3.48	
PLT (per mm^3^)		147	343.16 ± 92.24	
Lymphocyte%		148	36.20 ± 12.49	
Neutrophil%		148	54.45 ± 13.01	
Monocyte%		148	7.20 ± 2.76	
ESR (millimeters per hour)		130	15.39 ± 12.37	

Abbreviations: CRP, c‐reactive protein; CSF, cerebrospinal fluid; ESR, erythrocyte sedimentation rate; IVIG, intravenous immunoglobulin; MLR, monocyte‐lymphocyte ratio; NLR, neutrophil‐lymphocyte ratio; PLR, platelet‐lymphocyte ratio; PLT, platelet; WBC, white blood cells.

### 3.1 CBC ratios and GBS clinical features

Investigating the relation between inflammatory ratios and clinical presentation of GBS demonstrates that NLR and MLR were significantly higher in children with upper limb paralysis than patients without paralysis (*p* = 0.011 and 0.029, respectively), although no significant difference was seen based on PLR (Table 2).

**Table 2:** Mean and standard deviation of MLR, PLR, and NLR in patients according to clinical symptoms.

Clinical sign		Number	Mean	Standard deviation	*p* Value
NLR	Respiratory failure	6	3.20	2.57	0.224
	No respiratory failure	142	1.91	1.49	
	Upper limb paresis	74	2.22	1.72	**0.011**
	No upper limb paresis	74	1.70	1.33	
	Lower limb paresis	142	1.91	1.51	0.077
	No lower limb paresis	6	3.21	2.24	
	Inability to walk	139	1.98	1.59	0.764
	Ability to walk	9	1.75	0.76	
PLR	Respiratory failure	6	130.65	51.30	0.537
	No respiratory failure	141	123.97	67.07	
	Upper limb paresis	74	128.16	67.48	0.158
	No upper limb paresis	73	120.27	65.47	
	Lower limb paresis	141	122.45	65.24	0.117
	No lower limb paresis	6	166.3101	85.10650	
	Inability to walk	138	125.3116	67.94916	0.668
	Ability to walk	9	107.8504	31.84769	
MLR	Respiratory failure	6	0.2798	0.16604	0.409
	No respiratory failure	142	0.2433	0.24701	
	Upper limb paresis	74	0.2690	0.28478	**0.009**
	No upper limb paresis	74	0.2205	0.17780	
	Lower limb paresis	142	0.2384	0.23105	0.050
	No lower limb paresis	6	0.3952	0.35947	
	Inability to walk	139	0.2484	0.24477	0.939
	Ability to walk	9	0.1888	0.4583	

*Note*: *p* Value was measured based on Mann–Whitney *U* test. Bold values indicate statistically significant at *p* < 0.05.

Abbreviations: MLR, monocyte lymphocyte ratio; NLR, neutrophil lymphocyte ratio; PLR, platelet lymphocyte ratio.

### 3.2 CBC ratios and hospital stay

**Figure 2:**
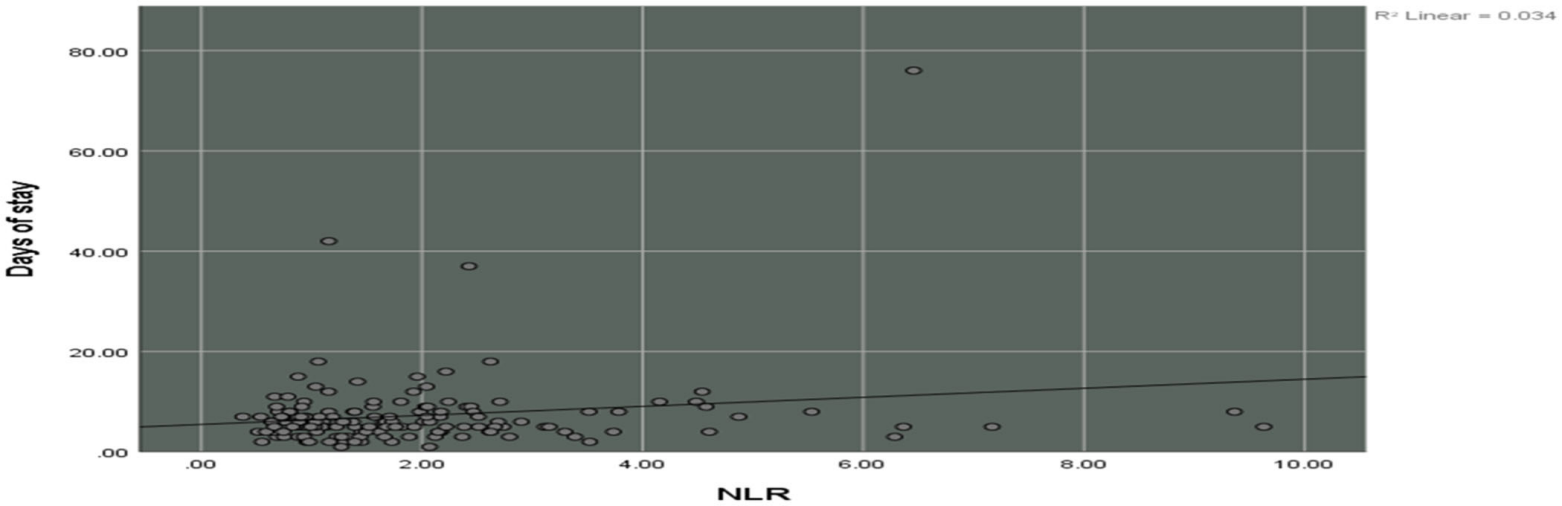
Simple scatter with the fit line of days of stay by the neutrophil‐lymphocyte ratio.

Our analysis revealed a direct correlation between length of hospital stay and all three inflammatory ratios NLR (coefficient = 0.184), MLR (coefficient = 0.098), and PLR (coefficient = 0.144), but the only statistically significant correlation was for NLR (*p* = 0.025) (Figure 2).

### 3.3 Electrodiagnostic study of GBS patients

One hundred twenty‐five patients underwent an EMG‐NCV study. Among them, 19 children had normal reports. The patterns of the EMG‐NCV study for 59 patients were reported as AMAN and 47 children showed AIDP pattern.

Electrodiagnostic data presented in Table 3 indicate that WBC is significantly different between the three different patterns of the EMG‐NCV study and its highest level is seen in the AIDP group and the lowest level in the normal EMG pattern (*p* = 0.015), also regarding CRP, a significant difference was seen between three groups (*p* = 0.025). Finally, a significant difference between the normal and abnormal pattern of EMG‐NCV was observed only based on CRP with *p* = 0.015.

**Table 3:** Laboratory data and length of hospital stay and age of GBS patients, according to the results of the electrodiagnostic study.

		Age	Hospital stay(days)	WBC	ESR	CRP	NLR	PLR	MLR
EMG‐NCV	AMAN	8 ± 4.2	7.5 ± 5.6	8.4 ± 2.2	16.4 ± 17.4	2.3 ± 3.1	1.6 ± 1.1	122.2 ± 63.3	0.21 ± 0.14
	AIDP	7.1 ± 3.6	7.8 ± 10.6	10.4 ± 4	12.8 ± 6.8	3.2 ± 7.5	2.1 ± 1.6	125.8 ± 54.3	0.21 ± 0.17
	NORMAL	7.8 ± 3	6 ± 3.1	9.6 ± 4.2	16.1 ± 13.6	7.5 ± 11.5	2.5 ± 2.3	143.8 ± 105.1	0.36 ± 0.50
*p* Value		0.614	0.427	**0.015**	0.803	**0.025**	0.368	0.735	0.442
EMG‐NCV	AMAN and AIDP	7.64 ± 3.98	7.70 ± 8.24	9.33 ± 3.29	14.82 ± 13.77	2.76 ± 5.58	1.88 ± 1.39	123.85 ± 59.31	0.21 ± 0.15
	Normal	7.8 ± 3	6 ± 3.1	9.6 ± 4.2	16.1 ± 13.6	7.5 ± 11.5	2.5 ± 2.3	143.8 ± 105.1	0.36 ± 0.50
*p* Value		0.619	0.314	0.889	0.605	**0.015**	0.694	0.960	0.227

*Note*: Bold values indicate statistically significant at *p* < 0.05.

Abbreviations: CRP, c‐reactive protein; ESR, erythrocyte sedimentation rate; MLR, monocyte‐lymphocyte ratio; NLR, neutrophil‐lymphocyte ratio; PLR, platelet‐lymphocyte ratio; WBC, white blood cells.

White blood cells (WBC), erythrocyte sedimentation rate (ESR), c‐reactive protein (CRP), neutrophil‐lymphocyte ratio (NLR), platelet lymphocyte ratio (PLR), and monocyte lymphocyte ratio (MLR), electromyography and nerve conduction studies (EMG‐NCV), acute motor axonal neuropathy (AMAN), acute inflammatory demyelinating polyneuropathy (AIDP). All data were reported as mean ± standard deviation and the *p* value was measured based on Kruskal–Wallis *H* Test.

The risk of ICU admission is 7.44 times higher in patients with abnormal patterns of electrodiagnostic study (AIDP and AMAN) than those with normal pattern (*p*: 0.027). Furthermore, significantly higher lower limb paralysis has been observed in the presence of abnormal EMG‐NCV results (*p*: 0.049). But there is no remarkable distinction between AIDP and AMAN patterns in terms of prognosis and clinical symptoms (Table 4).

**Table 4:** Clinical presentations of GBS patients according to the results of the electrodiagnostic study.

	AMAN + AIDP	NORMAL	*p* Value	AMAN	AIDP	*p* Value
Male	66	12	0.941	33	33	0.132
Female	40	7		26	14	
ICU admission	31	1	**0.027 (OR: 7.44)**	17	14	0.913
No ICU admission	75	18		42	33	
Respiratory failure	4	1	0.760	2	2	0.816
No respiratory failure	102	18		57	45	
Upper limp paresis	56	8	0.389	29	27	0.395
No upper limp paresis	50	11		30	20	
Lower limb paresis	104	2	**0.049 (OR: 6.118)**	59	45	0.110
No lower limp paresis	17	2		0	2	
Unable to walk	100	17	0.425	57	43	0.257
Able to walk	6	2		2	4	

*Note*: Bold values indicate statistically significant at *p* < 0.05.

Abbreviations: AIDP, acute inflammatory demyelinating polyneuropathy; AMAN, acute motor axonal neuropathy; ICU, intensive care unit; OR, odds ratio.

### 3.4 Cerebrospinal fluid analysis

Lumbar puncture was conducted on 46 patients, and the mean and standard deviation (SD) of time between the onset of symptoms and the procedure was 2 ± 5 days (range: 1–25 days). Among children who underwent cerebrospinal fluid analysis, 89% had elevated levels of CSF protein. The mean and SD of WBC in Guillain–Barre syndrome patients was 5.2 + 12.1 per mm^3^. Of the 46 individuals, 34 (74%) had ACD patterns in their CSF test results, while the other 12 showed different patterns than ACD. The study revealed that NLR, MLR, and PLR are related to the ACD pattern, so in ACD pattern patients, these three inflammatory markers were significantly higher than other patterns (*p* = 0.002, 0.021, and 0.031, respectively). No significant correlation was found in other situations. Moreover, individuals with ACD patterns were 1.5 times more likely to be admitted to ICU, according to the data (Tables 5 and 6).

**Table 5:** Laboratory data and length of hospital stay and age of GBS patients, according to the results of the cerebrospinal fluid analysis.

	CSF pattern	Number	Mean ± standard deviation	*p* Value
Age	ACD	34	7.5588 ± 4.01657	0.435
	Other	12	6.4167 ± 3.57919	
Hospital stays (days)	ACD	34	10.2647 ± 13.21915	0.763
	Other	12	7.1667 ± 2.51661	
WBC	ACD	34	9.7568 ± 4.38703	0.157
	Other	12	8.3267 ± 2.73990	
PLT	ACD	34	348.5882 ± 91.36710	0.083
	Other	12	296.5000 ± 73.95023	
ESR	ACD	30	17.5667 ± 19.96667	0.232
	Other	11	9.9091 ± 5.24318	
CRP	ACD	26	2.5769 ± 5.44921	0.965
	Other	9	5.7778 ± 10.98610	
NLR	ACD	34	2.0324 ± 1.23041	**0.002**
	Other	12	1.1715 ± 1.13825	
PLR	ACD	34	123.9654 ± 51.15229	**0.021**
	Other	12	100.5437 ± 83.33149	
MLR	ACD	34	0.2463 ± 0.20356	**0.031**
	Other	12	0.2199 ± 0.23259	

*Note*: *p* Value regarding PLT was measured based on an Independent *t* test, and Mann–Whitney *U* test was conducted for other variables. Bold values indicate statistically significant at *p* < 0.05.

Abbreviations: ACD, albuminocytological dissociation; CRP, c‐reactive protein; CSF, cerebrospinal fluid; ESR, erythrocyte sedimentation rate; MLR, monocyte‐lymphocyte ratio; NLR, neutrophil‐lymphocyte ratio; PLR, platelet‐lymphocyte ratio; PLT, platelets; WBC, white blood cells.

**Table 6:** Clinical presentations of GBS patients according to the results of the cerebrospinal fluid analysis.

	ACD	Other	*p* Value
Male	25	7	0.325
Female	9	5	
ICU admission	10	0	**0.034 (OR: 1.5)**
No ICU admission	24	12	
Respiratory failure	4	0	0.214
No respiratory failure	30	12	
Upper limp paresis	17	7	0.619
No upper limp paresis	17	5	
Lower limb paresis	32	12	0.390
No lower limp paresis	2	0	
Unable to walk	32	9	0.067
Able to walk	2	3	

Abbreviations: ACD, albuminocytological dissociation; ICU, intensive care unit; OR, odds ratio.

### 3.5 Prognostic factors for ICU admission

Binary logistic regression was performed to determine the prognostic risk factors of ICU admission (due to the small sample size, CSF results were not included). Only respiratory failure at the time of admission significantly (*p* = 0.021) increased the possibility of ICU admission by 17.993 times, and none of the other factors were predictive for ICU admission.

### 3.6 COVID‐19 pandemic

Eighty‐seven patients in our study had been admitted previous to the onset of the COVID‐19 pandemic in Iran. Following the pandemic, an additional 63 GBS patients were admitted. Our analysis revealed significant differences between these two groups in terms of laboratory results, specifically WBC and ESR (*p* = 0.020 and 0.006 respectively). The most noteworthy finding was related to the EMG‐NCV study, which showed a significantly higher incidence of the normal pattern of the Electrodiagnostic study after the pandemic (*p* = 0.011). Surprisingly, before the pandemic, AIDP was the leading pattern of EMG‐NCV, but after the pandemic, the AMAN pattern was the most prevalent in GBS patients (*p* < 0.001). In addition, there were significant differences in clinical presentation, particularly lower limb paresis and inability to walk with a *p* value of 0.033 and 0.008 respectively (Tables 7 and 8).

**Table 7:** Binary logistic regression regarding ICU admission.

	Independent variables	*p* Value	Odds ratio
ICU admission	Sex	0.068	0.376
	Age	0.061	1.134
	NLR	0.752	1.076
	PLR	0.187	0.993
	MLR	0.421	3.853
	Respiratory failure	**0.035**	17.993
	Upper limp paresis	0.115	2.178
	Lower limb paresis	0.970	1.101
	Unable to walk	0.999	519765899.7
	Abnormal pattern of EMG‐NCV (AMAN, AIDP)	0.097	9.436

*Note*: Bold value indicate statistically significant at *p* < 0.05.

Abbreviations: AIDP, acute inflammatory demyelinating polyneuropathy; AMAN, acute motor axonal neuropathy; ICU, intensive care unit; MLR, monocyte‐lymphocyte ratio; NLR, neutrophil‐lymphocyte ratio; PLR, platelet‐lymphocyte ratio.

**Table 8:** Laboratory data and length of hospital stay and age of GBS patients, based on admission date.

	Date	Number	Mean ± standard deviation	*p* Value
Age (years)	Before COVID‐19	87	7.3793 ± 3.68921	0.701
	After COVID‐19	63	7.7460 ± 3.86034	
Hospital stay (days)	Before COVID‐19	87	7.4483 ± 8.28386	0.531
	After COVID‐19	63	6.9365 ± 6.43547	
CSF protein	Before COVID‐19	23	81.3043 ± 63.15237	0.852
	After COVID‐19	23	90.3913 ± 70.28820	
CSF WBC	Before COVID‐19	23	4.5217 ± 6.68016	0.541
	After COVID‐19	23	6.0000 ± 15.95733	
WBC	Before COVID‐19	85	10.0180 ± 4.03265	**0.020**
	After COVID‐19	63	8.6618 ± 2.37015	
PLT	Before COVID‐19	84	351.7500 ± 96.08642	0.193
	After COVID‐19	63	331.7143 ± 86.27571	
ESR	Before COVID‐19	74	16.8649 ± 13.76647	**0.006**
	After COVID‐19	56	13.4357 ± 12.68953	
CRP	Before COVID‐19	64	5.7188 ± 11.24259	0.632
	After COVID‐19	53	2.1887 ± 1.80870	
PLR	Before COVID‐19	84	126.9227 ± 69.48852	0.742
	After COVID‐19	63	120.6691 ± 62.35243	
MLR	Before COVID‐19	85	0.2682 ± 0.29703	0.961
	After COVID‐19	63	0.2131 ± 0.11314	
NLR	Before COVID‐19	85	2.0960 ± 1.60128	0.189
	After COVID‐19	63	1.7845 ± 1.48355	

*Note*: *p* Value regarding PLT was measured based on an Independent *t* test, and Mann–Whitney *U* Test was conducted for other variables.

Abbreviations: CRP, C‐reactive protein; CSF, cerebrospinal fluid; ESR, erythrocyte sedimentation rate; MLR, monocyte‐lymphocyte ratio; NLR, neutrophil‐lymphocyte ratio; PLR, platelet‐lymphocyte ratio; PLT, platelets; WBC, white blood cells.

## 4 DISCUSSION

In our study, male patients accounted for 61.6% of all patients, which is compatible with the results of previously published studies, as males have a higher incidence than females.^21^, ^22^

Also, congruently to former studies, 61% of our patients reported having an antecedent respiratory or gastrointestinal viral infection before the onset of GBS symptoms.^2^

According to our study, 4%, 50%, 96%, and 94% of GBS patients presented with symptoms of respiratory failure, upper limb paralysis, lower limb paralysis, and inability to walk, respectively. Similar to other studies, limb weakness was the most common presentation.^23^, ^24^

The mean of PLR, NLR, and MLR in our patients have been 124.2426, 1.9634, and 0.2448, respectively, which are consistent with the study of Kim and colleagues, which reported the average PLR and NLR in fully recovered patients to be 112.7 and 1.38, respectively.^25^ Ethemoglu and colleagues reported that NLR in hospitalized, discharged and control participants were 1.51, 1.47, and 1.39, respectively. In consonance with the same study, PLR in hospitalized, discharged and control subjects was 114.81, 110.7, and 95.09 respectively.^11^ Additionally, as stated by Huner and colleagues NLR was 3.29 and 1.27 in subjects enrolled in the study before and after IVIG treatment. Also, according to this study, PLR in the subjects before and after receiving IVIG was 126.74 and 127.5.^26^

We found that NLR can be used potentially as a prognostic and diagnostic factor in children with GBS. In our patients, NLR had a statistically significant difference between people with and without upper limb paralysis. Also, an increase in NLR significantly increases the length of hospital stay. Some past studies also confirm our results.^11^, ^12^, ^19^ However, studies such as Ozdemir and colleagues showed that there is no correlation between NLR, PLR, and the prognosis of children with GBS.^13^

Patients who showed ACD pattern in CSF analysis had significantly higher levels of NLR, MLR, and PLR. ACD pattern in CSF has a meaningful correlation with ICU stay (Table 5).

A previous study in Turkey showed that in adult GBS patients higher CRP, NLR, and PLR were associated with severe disability and worse prognosis of GBS.^27^ Additionally, it is reported that a higher level of CSF protein is correlated with a worse prognosis.^28^

Analysis of the electrodiagnostic study illustrated that the risk of admission to the ICU and also lower limb paralysis is significantly higher in the abnormal EMG‐NCV pattern, furthermore, WBC is significantly higher in the AIDP group. The outcome was the same in all patterns of the EMG‐NCV. The result of the study of Tekgul and colleagues, was almost compatible with ours, as the final result was the same between the demyelinating group and the axonal involvement form, but the response to treatment was faster in the demyelinating group.^8^

The most common type of electrophysiology pattern in GBS is AIDP.^29^ During the COVID‐19 pandemic, the AMAN pattern was the most prevalent electrophysiologic result in our GBS patients (Table 9) and the level of WBC and ESR were significantly lower than the pre‐COVID‐19 pandemic (Table 8).

**Table 9:** Clinical presentations of GBS patients based on the admission date.

	After COVID‐19	Before COVID‐19	*p* Value
Male	42	51	0.316
Female	21	36	
ICU admission	19	19	0.248
No ICU admission	44	68	
Respiratory failure	4	2	0.212
No respiratory failure	59	85	
Upper limb paresis	30	45	0.620
No upper limb paresis	33	42	
Lower limb paresis	63	81	**0.033**
No lower limb paresis	0	6	
Unable to walk	63	78	**0.008**
Able to walk	0	9	
AMAN + AIDP	50	56	**0.011**
Normal EMG‐NCV pattern	3	16	
AMAN	41	18	**0.000**
AIDP	9	38	
ACD pattern in LP	19	15	0.179
Other patterns in LP	4	8	

*Note*: *p* Value was measured based on Fisher's Exact test. Bold values indicate statistically significant at *p* < 0.05.

Abbreviations: ACD, albuminocytological dissociation; AIDP, acute inflammatory demyelinating polyneuropathy; AMAN, acute motor axonal neuropathy; EMG‐NCV, electromyography and nerve conduction velocity; ICU, intensive care unit; LP, lumbar puncture.

We found that the number of white blood cells had been significantly higher in the GBS patients who had an AIDP pattern and it was lowest in those with normal electrophysiology study (Table 3). There wasn't any significant difference in the presentation and prognosis of those with AMAN and AIDP types (Table 4). However, in a study performed in Iran, the axonal subtype was correlated with a worse prognosis.^30^

In studies mainly conducted in adults, several factors have been suggested as prognostic elements for GBS including; severe onset, cranial nerve involvement, autonomic nerve involvement, need for mechanical ventilation, previous diarrhea, short interval between onset of symptoms and admission, axonal lesion patterns in the electrophysiological study and older age.^31^, ^32^, ^33^ However, in young children, a delay in achieving the ability to walk independently was associated with poor prognosis.^34^

We found that ICU admission had a significant relation with respiratory failure and it wasn't related to the clinical symptoms, age, sex, and laboratory indicators.

Overall, GBS is a life‐threatening disease with a mortality rate of 7%–11% in children. Patients die mainly from respiratory failure, pulmonary complications, or autonomic dysfunction. Those who survive are often affected by residual complaints and deficits that significantly affect their daily activities and quality of life. Improvements occur mostly within a year after the onset of GBS, although patients may experience further improvement even after 3 years or more.^3^, ^25^, ^26^ In our study, no mortality was seen and all patients were discharged.

The COVID‐19 pandemic has impacted and altered the characteristics of numerous diseases.^35^, ^36^, ^37^ Our study revealed that the pandemic has changed the most frequent EMG‐NCV pattern from AIDP to AMAN in Iranian patients with GBS. This intriguing discovery necessitates validation, and further research is required to elucidate the cellular mechanism behind this issue.

### 4.1 Limitations

In the current study, the standard functional and disability scores for the classification of the patients had not been recorded and the autonomic dysfunction had not been evaluated. Longer follow‐up for assessing the disabilities could provide better evidence of the nature of the disease that could be considered in future studies. Future studies with larger sample size and longer follow‐ups to investigate the results of the present study is recommended.

## 5 CONCLUSION

We conducted a study on Iranian children with GBS, analyzing their clinical, laboratory, and electrodiagnostic results. Our findings indicate that there may be a relationship between GBS pathogenesis and NLR, as higher NLR levels were associated with longer hospital stays and upper limb paralysis. Additionally, we found that abnormal EMG patterns were useful to predict GBS prognosis, with a higher likelihood of ICU admission and lower limb paralysis. Our research also revealed that the COVID‐19 pandemic has affected the typical EMG‐NCV pattern in Iran, highlighting the need for further investigation.

## AUTHOR CONTRIBUTIONS

**Sepide Javankiani**: Conceptualization; data curation; investigation; writing—original draft; writing—review & editing. **Amir Nasrollahizadeh**: Formal analysis; methodology; writing—original draft; writing—review & editing. **Behdad Gharib**: Data curation; investigation; writing—original draft. **Morteza Heidari**: Data curation; investigation; project administration; resources. **Sara Memarian**: Conceptualization; data curation; project administration; supervision.

## CONFLICT OF INTEREST STATEMENT

The authors declare no conflict of interest.

## ETHICS STATEMENT

This study was approved by the Institutional Review Board (IRB) of Tehran University of Medical Sciences, Tehran with the ethics code “IR.TUMS.CHMC.REC.1399.125.”

## TRANSPARENCY STATEMENT

The lead author Sara Memarian affirms that this manuscript is an honest, accurate, and transparent account of the study being reported; that no important aspects of the study have been omitted; and that any discrepancies from the study as planned (and, if relevant, registered) have been explained.

## Data Availability

The data set is not publicly available. Requests to access these data sets should be directed to the corresponding author, Sara.memarian64@gmail.com
